# The effects on calcaneofibular ligament function of differences in the angle of the calcaneofibular ligament with respect to the long axis of the fibula: a simulation study

**DOI:** 10.1186/s13047-017-0242-1

**Published:** 2017-12-28

**Authors:** Mutsuaki Edama, Ikuo Kageyama, Takanori Kikumoto, Masatoshi Nakamura, Wataru Ito, Emi Nakamura, Ryo Hirabayashi, Tomoya Takabayashi, Takuma Inai, Hideaki Onishi

**Affiliations:** 1 Institute for Human Movement and Medical Sciences, Niigata University of Health and Welfare, Shimami-cho 1398, Kita-ku, Niigata City, 950-3198 Japan; 2 Department of Anatomy, School of Life Dentistry at Niigata, Nippon Dental University, Niigata, Japan

**Keywords:** Lateral ankle ligament injury, Ankle inversion restriction, Lateral ankle ligament complex

## Abstract

**Background:**

In the present study, CFLs harvested from cadavers were categorized according to the differences in the angle of the CFL with respect to the long axis of the fibula and their shape, and then three-dimensional reconstructions of the CFLs were used to simulate and examine the differences in the angles of the CFLs with respect to the long axis of the fibula and how they affect CFL function.

**Methods:**

The study sample included 81 ft from 43 Japanese cadavers. CFLs were categorized according to their angle with respect to the long axis of the fibula and the number of fiber bundles. Five categories were subsequently established: CFL20° (angle of the CFL with respect to the long axis of the fibula from 20° to 29°); CFL30° (range 30–39°); CFL40° (range 40–49°); CFL50° (range 50–59°); and CFL2 (CLFs with two crossing fiber bundles). Three-dimensional reconstructions of a single specimen from each category were then created. These were used to simulate and calculate CFL strain during dorsiflexion (20°) and plantarflexion (30°) on the talocrural joint axis and inversion (20°) and eversion (20°) on the subtalar joint axis.

**Results:**

In terms of proportions for each category, CFL20° was observed in 14 ft (17.3%), with CFL30° in 22 ft (27.2%), CFL40° in 29 ft (35.8%), CFL50° in 15 ft (18.5%), and CFL2 in one foot (1.2%). Specimens in the CFL20° and CFL30° groups contracted with plantarflexion and stretched with dorsiflexion. In comparison, specimens in the CFL40°, CFL50°, and CFL2 groups stretched with plantarflexion and contracted with dorsiflexion. Specimens in the CFL20° and CFL2 groups stretched with inversion and contracted with eversion.

**Conclusions:**

CFL function changed according to the difference in the angles of the CFLs with respect to the long axis of the fibula.

## Background

Many studies have investigated the functional role of the CFL in addition to the ATFL because the ATFL and CFL are largely involved in ankle inversion restriction [1, 2]. However, consensus has yet to be reached on the functional role of the CFL.

For example, Ozeki et al. examined cadavers to explore the functional role of the CFL and observed that the CFL becomes taut at dorsiflexion angles ≥18°, and it is nearly relaxed at other angles [3]. Furthermore, using simulation models created from experiments with cadavers, Leardini et al. attempted to predict the roles of individual ligaments at various flexion angles using the effective length fraction, which is the ratio between the measured length of the ligament and the length of the ligament at maximum elongation [4]. From the observation that changes in ligament length during ankle flexion and extension were slight, this study argued that it is very likely that the CFL plays a major role in stabilizing ankle flexion and extension. However, although Sarrafian and Kelikian found that the CFL is taut in dorsiflexion and relaxed in plantarflexion, they also noted that some specimens showed reversal of motions, whereas, in others, the tension in this ligament remained constant in all positions [5]. It has been suggested that this may be due to the differences in the angle of the CFL with respect to the long axis of the fibula [3, 5]. In addition, it has also been indicated that restrictions on ankle inversion and eversion vary depending on the running angle [5]. Nevertheless, these and other studies relied on observations, and there has been no research into the effects on ankle function of the differences in the angle of the CFL with respect to the long axis of the fibula.

Analyzing data from autopsies and operations, Ruth identified four categories of CFLs based on angles relative to the long axis of the fibula and morphology: 0° (14 ft, 18.7%), 10–45° (56 ft, 74.7%), 80–90° (three feet, 4%), and fan-shaped CFLs (two feet, 2.6%) [6]. Kitsoulis found 52 subjects (72.2%) with one band forming the CFL, 16 with two bands (22.2%), and 4 with three bands (5.6%) [7].Other studies have investigated morphological characteristics of the CFL such as thickness, width, and length [8–11], but there has been no research into the effects of the differences in the angle of the CFL with respect to the long axis of the fibula and morphologies on ankle function.

Therefore, In the present study, CFLs harvested from cadavers were categorized according to the differences in the angle of the CFL with respect to the long axis of the fibula and the shape, and then three-dimensional reconstructions of the CFLs were used to simulate and examine the differences in the angles of the CFLs with respect to the long axis of the fibula and how they affect CFL function.

## Methods

### Cadavers

A total of 81 legs from 43 Japanese cadavers (mean age at death, 77 ± 12 years; 51 sides from men, 30 from women; 41 sides from right, 40 sides from left) that had been switched to alcohol after placement in 10% formalin were examined. None showed signs of previous major surgery around the ankle. This study was approved by the Ethics Committee at our institution.

### Methods

**Fig. 1:**
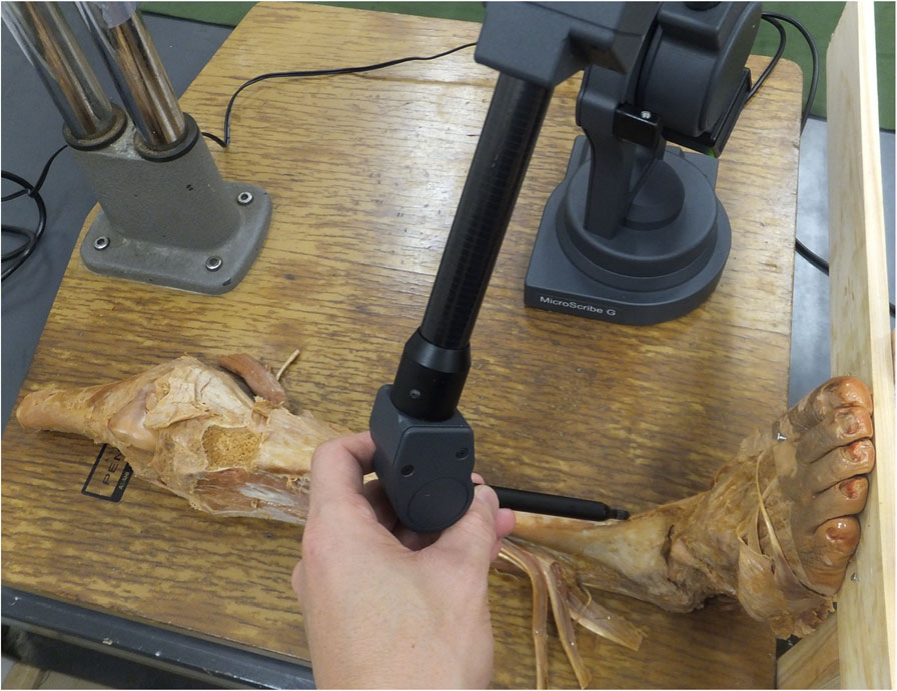
The MicroScribe system

One author (first author) dissected the CFL ligaments alone. The lower limbs were cut 10 cm above the knee to produce isolated specimens. The CFLs were carefully dissected after removal of skin, subcutaneous tissue, muscle-tendon tissue, and crural fascia. With reference to a previous study [6], the CFLs were categorized according to the angles of the CFLs with respect to the long axis of the fibula and the number of fiber bundles, using a stainless 180 goniometer (300 mm CK-S4305-300, Chin Kou Medical Instrument Ltd., New Taipei City**,** Taiwan). Five categories were established: CFL20° (angle of the CFL with respect to the long axis of the fibula from 20° to 29°); CFL30° (range 30–39°); CFL40° (range 40–49°); CFL50° (range 50–59°); and CFL2 (CFLs with two crossing fiber bundles). All measurements were carefully done in an intermediate position of ankle plantarflexion/dorsiflexion of 0° and foot inversion/eversion of 0°. A single specimen from each category was then selected, and three-dimensional reconstructions were created from five feet, using the MicroScribe system (G2X-SYS, Revware, NC, USA) to digitize two points, the origin and terminus, of the CFL (Fig. 1). The Rhinoceros 3D software program (McNeel, Seattle, WA, USA) was used to construct the three-dimensional models. The talocrural joint (the line connecting the inferior borders of the medial and lateral malleoli) and the subtalar joint (the line connecting the lateral border of the calcaneal tuberosity and the midpoint of the talar head) were designated as the joint axes [12–15]. The simulations were then used to calculate CFL strain (%) during dorsiflexion (20°) and plantarflexion (30°) on the talocrural joint axis and during inversion (20°) and eversion (20°) on the subtalar joint axis. Using the following formula, CFL strain was expressed as the percentage of change of ligament length from the initial limb position (LTS), when both flexion/extension and inversion/eversion were 0°, to the final position (LT) after motion [16].

$$ Strain\left(\%\right)=\left[\left(\frac{L^T-{L}_S^T}{L_S^T}\right)\times 100\right] $$

The MicroScribe system is an instrument with high precision (manufacturer’s specifications, measurement precision of 0.23 mm). However, measurements must be performed manually. In addition, although the study cadavers were thoroughly fixed to the examination table such that they did not move, it was necessary to test whether they had moved, since the measurements entail dissection of the ligament tissue. A previous study by the authors found the intraclass correlation coefficient (1, 1) to be 0.97–0.99 [17], which indicates a high level of reliability and reproducibility.

### Statistical analysis

A chi-squared test with a significance level of 5% was used to compare CFL running angle differences between men and women and between left and right feet.

## Results

### CFL running angle categories (Fig. 2)

Five categories were identified: CFL20° in 14 ft (17.3%); CFL30° in 22 ft (27.2%); CFL40° in 29 ft (35.8%); CFL50° in 15 ft (18.5%); and CFL2 in one foot (1.2%).

**Fig. 2:**
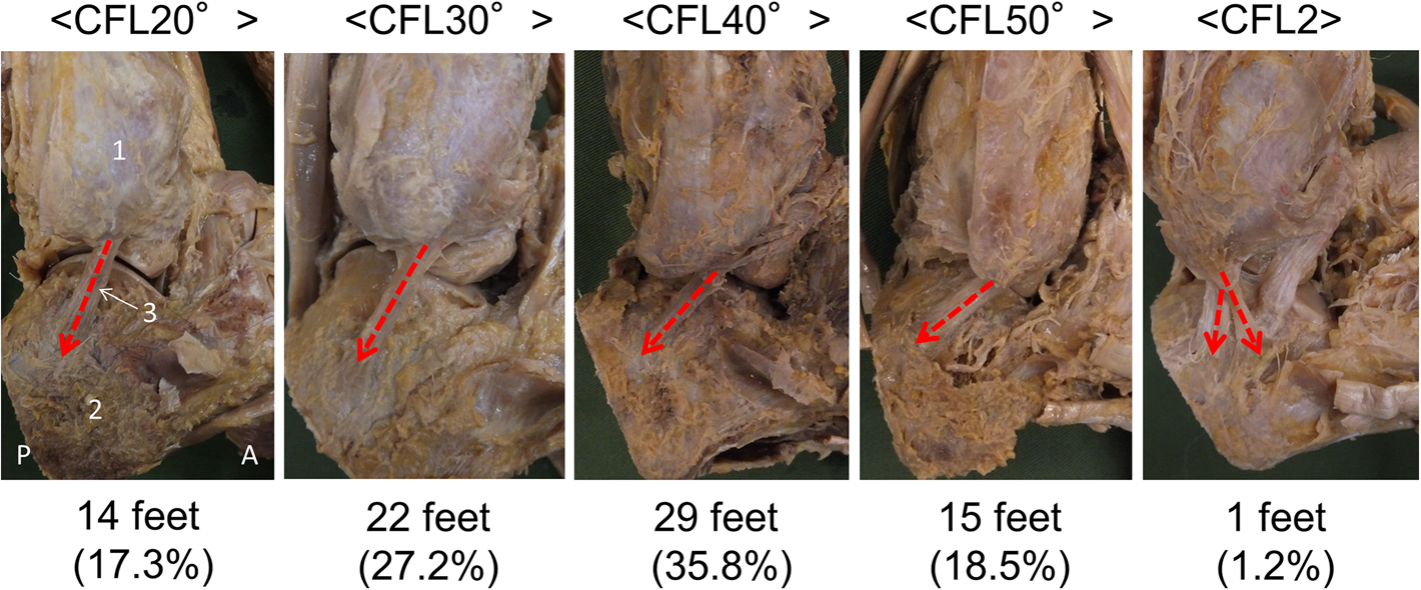
Classification of calcaneofibular ligaments by running angle, right foot, lateral view. CFL20°, angle of the CFL with respect to the long axis of the fibula from 20° to 29°; CFL30°, angle of the CFL with respect to the long axis of the fibula from 30° to 39°; CFL40°, angle of the CFL with respect to the long axis of the fibula from 40° to 49°; CFL50°, angle of the CFL with respect to the long axis of the fibula from 50° to 59°; CFL2, CFLs with two crossing fiber bundles. 1: Fibula, 2: Calcaneus, 3: Calcaneofibular ligament, A: Anterior, P: Posterior

### Sex and left-right differences

A comparison between males and females showed that, among men, 7 legs (8.6%) were CFL20°, 13 ft (16.1%) were CFL30°, 18 ft (22.2%) were CFL40°, 13 ft (16.1%) were CFL50°, and 0 ft (0%) were CFL2, while, among women, 7 ft (8.6%) were CFL20°, 9 ft (11.1%) were CFL30°, 11 ft (13.6%) were CFL40°, 2 ft (2.5%) were CFL50°, and 1 ft (1.2%) was CFL2. No significant differences were seen (*p* = 0.101).

With respect to left-right differences, both legs could be measured in 37 cadavers (male 23, female 14; 74 ft). Among the right feet, 5 ft (6.7%) were CFL20°, 9 ft (12.2%) were CFL30°, 15 ft (20.3%) were CFL40°, 8 ft (10.8%) were CFL50°, and 0 ft (0%) were CFL2. Among the left feet, 7 ft (9.4%) were CFL20°, 11 ft (14.9%) were CFL30°, 11 ft (14.9%) were CFL40°, 7 ft (9.4%) were CFL50°, and 1 ft (1.4%) was CFL2. No significant differences were seen (*p* = 0.665).

### Changes in strain for each category during dorsiflexion and plantarflexion (Table 1)

The mean change in strain for all categories ranged from -1.3% to 0.3%. Specimens in the CFL20° and CFL30° groups contracted with plantarflexion and stretched with dorsiflexion. In comparison, specimens in the CFL40°, CFL50°, and CFL2 groups stretched with plantarflexion and contracted with dorsiflexion.

**Table 1:** Changes in strain for each category during dorsiflexion and plantarflexion

	Plantarflexion				Dorsiflexion	
	30°	20°	10°	0°	10°	20°
CFL20°	−4.5	−3.0	−1.4	0	1.3	2.4
CFL30°	−6.4	−3.7	−1.6	0	1.0	1.5
CFL40°	3.0	2.2	1.2	0	−1.4	−2.9
CFL50°	7.0	5.1	2.7	0	−3.1	−6.5
CFL2	2.5	1.6	0.8	0	−0.7	−1.2
Average	0.3	0.4	0.3	0	−0.6	−1.3

Value: Strain at plantarflexion and dorsiflexion

CFL20°, angle of the CFL with respect to the long axis of the fibula from 20° to 29°; CFL30°, angle of the CFL with respect to the long axis of the fibula from 30° to 39°; CFL40°, angle of the CFL with respect to the long axis of the fibula from 40° to 49°; CFL50°, angle of the CFL with respect to the long axis of the fibula from 50° to 59°; CFL2, CFLs with two crossing fiber bundles

### Changes in strain when ankle varus (20°) and valgus (20°) were added at the ankle joint with plantarflexion/dorsiflexion for each CFL category (Table 2)

The means for all categories showed that the sample as a whole tended to stretch with inversion and contract with eversion in both dorsiflexion and plantarflexion. Furthermore, specimens showed more elongation when inverted during plantarflexion than during dorsiflexion. Specimens in the CFL20° and CFL2 groups stretched with inversion and contracted with eversion.

**Table 2:** Changes in strain when ankle varus (20°) and valgus (20°) were added at the ankle joint with plantarflexion/dorsiflexion for each calcaneofibular ligament category

	Plantarflexion				Dorsiflexion	
	30°	20°	10°	0°	10°	20°
CFL20°	3.7 / -10.1	5.2 / -8.6	6.7 / -7.0	8.1 / -5.6	9.4 / -4.2	10.5 / -3.0
CFL30°	−6.1 / 5.0	−3.7 / -2.1	−1.8 / 0.3	−0.5 / 2.1	0.3 / 3.4	0.4 / 4.1
CFL40°	2.9 / 6.5	2.0 / 5.8	0.8 / 5.0	−0.5 / 3.8	−2.0 / 2.5	−3.6 / 1.0
CFL50°	8.0 / 7.7	5.9 / 6.0	3.4 / 3.8	0.4 / 1.3	−2.8 / -1.7	−6.3 / -4.9
CFL2	14.1 / -2.4	13.2 / -3.3	12.5 / -4.2	11.8 / -5.1	11.2 / -5.9	10.8 / -6.5
Average	4.5 ± 7.4 / -0.7 ± 7.6	4.5 ± 6.2 / -0.4 ± 6.3	4.3 ± 5.5 / -0.4 ± 5.1	3.9 ± 5.7 / -0.7 ± 4.3	3.2 ± 6.6 / -1.2 ± 4.1	2.4 ± 7.9/ -1.9 ± 4.4

Value: Strain at Inversion/Strain at Eversion

Average: Values presented as means ± SD

CFL20°, angle of the CFL with respect to the long axis of the fibula from 20° to 29°; CFL30°, angle of the CFL with respect to the long axis of the fibula from 30° to 39°; CFL40°, angle of the CFL with respect to the long axis of the fibula from 40° to 49°; CFL50°, angle of the CFL with respect to the long axis of the fibula from 50° to 59°; CFL2, CFLs with two crossing fiber bundles

## Discussion

The present study attempted to elucidate the effects of CFL running angle and morphology on ankle function by categorizing CFLs from cadavers according to these characteristics and conducting simulations using three-dimensional reconstructions. To the best of our knowledge, there have been no anatomical or biomechanical studies focusing on the relationships between ankle function and CFL running angle and morphology.

In the present study, five categories were identified. The present sample comprised 65 ft (80.3%) with an angle respect to the long axis of the fibula between 10° and 50°, which was similar to the finding of 56 ft (74.7%) reported in the previous study [6]. Unlike the previous study, though, the present sample did not include specimens with fan-shaped morphology or with an angle with respect to the long axis of the fibula of 0° and 80–90° [6] or with three bands [7].

The mean change in strain for all categories during ankle flexion and extension ranged from −1.3% to 0.3%, indicating that the CFL was not stretched for the most part. Previous studies using cadavers found that, during dorsiflexion and plantarflexion, the CFL was nearly relaxed [3] or did not change significantly in length [4], and it has been proposed that such observations suggest that the CFL is highly involved in stabilizing ankle flexion and extension [4]. Likewise, the present study obtained similar results. Although Sarrafian and Kelikian found that the CFL is taut during both dorsiflexion and plantarflexion, they also noted that some joints showed reversal of motions, while other joints displayed no change in tautness [5]. The present study detected contraction in plantarflexion and elongation in dorsiflexion for specimens in the CFL20° and CFL30° categories. In comparison, specimens in the CFL40°, CFL50°, and CFL2 categories showed elongation in plantarflexion and contraction in dorsiflexion. These results indicate that CFL strain in plantarflexion and dorsiflexion may vary depending on the CFL running angle.

In regards to changes in strain for each category when 20° inversion and 20° eversion were applied during ankle flexion and extension, the means for all categories showed that the sample as a whole stretched with inversion in both dorsiflexion and plantarflexion. Past studies with frozen cadavers reported that the CFL is highly involved in limiting ankle inversion [1, 2]. The results of the present study correspond with this view. Although the means for all categories demonstrated that the CFL tends to stretch when inversion is applied in plantarflexion as compared to dorsiflexion, CFL strain values were uneven among individual categories. The results of past studies have been inconsistent on this point. For example, one study found that the CFL is stretched with ankle inversion [5], while another study reported that maximal CFL elongation occurs with pronation-external rotation of the foot during plantarflexion [18]. Such findings suggest the possibility that differences in the angle of the CFL with respect to the long axis of the fibula may be an influential factor.

In the present study, at 0° ankle flexion and extension, specimens displayed elongation with inversion and contraction with eversion, especially in the CFL20° and CFL2 categories. Sarrafian and Kelikian observed that, because the CFL terminus plane of motion is perpendicular to the subtalar joint axis of motion when the ankle is in the neutral position, the CFL will contract with subtalar inversion and stretch with subtalar eversion when the running angle is perpendicular to the fibula and show the exact opposite tendency when the angle of the CFL with respect to the long axis of the fibula is parallel to the fibula [5]. Accordingly, this appears to be the reason why specimens in the CFL20° category, which were similar to the angle of the CFL with respect to the long axis of the fibula, stretched with inversion and contracted with eversion.

This study did have a number of limitations. First, it involved simulations with cadavers. Therefore, gravity, weight bearing, muscle activity, and the posture of the foot were not considered. In the future, we believe that it will be necessary to perform biomechanical research using our basic data with in vivo samples. Second, all cadavers used in this study were Japanese. It is not certain whether the present findings apply to cadavers from other ethnicities. Many studies have raised the possibility of skeletal muscle and tendon variations across ethnicities [19–23], and this could be true for ligaments as well. Thus, future studies will need to investigate variations based on ethnic origin.

## Conclusions

The findings of the present study suggest that CFL function may change in relation to the angle of the CFL with respect to the long axis of the fibula and morphologies. In the future, we believe that it will be necessary to perform biomechanical research using our basic data with in vivo samples which may lead to the elucidation of the functional role of CFL. Furthermore, we believe that it would be valuable to confirm whether individual anatomical differences may represent risk factors for CFL injury.
